# Access to Bacteriologic-Based Diagnosis in Smear Positive Retreatment Tuberculosis Patients in Rural China: A Cross-Sectional Study in Three Geographic Varied Provinces

**DOI:** 10.1371/journal.pone.0146340

**Published:** 2016-01-11

**Authors:** Changming Zhou, Weili Jiang, Li Yuan, Wei Lu, Jinge He, Qi Zhao, Biao Xu

**Affiliations:** 1 Department of Epidemiology, School of Public Health, Fudan University, Shanghai, China; 2 Key Laboratory of Public Health Safety (Ministry of Education), Shanghai, China; 3 Jiangsu Provincial Center for Disease Control, Nanjing, China; 4 Sichuan Provincial Center for Disease Control, Chengdu, China; 5 Centre for Global Health, Karolinska institutet, Stockholm, Sweden; Public Health Research Institute at RBHS, UNITED STATES

## Abstract

**Objective:**

To determine factors influencing the utilization and accessibility to bacteriologic-based tuberculosis (TB) diagnosis among sputum smear positive (SS+) retreatment TB patients, and to develop strategies for improving the case detection rate of MDR-TB in rural China.

**Study Design and Setting:**

A cross-sectional study of SS+ TB retreatment patients was conducted in eight counties from three provinces with different implementation period and strategy of MDR-TB program in China. Demographic and socioeconomic parameters were collected by self-reporting questionnaires. Sputum samples were collected and cultured by the laboratory of county-designated TB clinics and delivered to prefectural Centers for Disease Prevention and Control (CDC) labs for DST with 4 first-line anti-TB drugs.

**Results:**

Among the 196 SS+ retreatment patients, 61.22% received culture tests during current treatment. Patients from more developed regions (OR = 24.0 and 3.6, 95% CI: 8.6–67.3 and 1.1–11.6), with better socio-economic status (OR = 3. 8, 95% CI: 1.3–10.7), who had multiple previous anti-TB treatments (OR = 5.0, 95% CI: 1.6–15.9), and who failed in the most recent anti-TB treatment (OR = 2.6, 95% CI: 1.0–6.4) were more likely to receive culture tests. The percentage of isolates resistant to any of first-line anti-TB drugs and MDR-TB were 50.0% (95% CI: 39.8%-60.2%) and 30.4% (95% CI: 21.0%-39.8%) respectively.

**Conclusions:**

Retreatment SS+ TB patients, high risk MDR-TB population, had poor utilization of access to bacteriologic-based TB diagnosis, which is far from optimal. The next step of anti-TB strategy should be focused on how to make bacteriological-based diagnosis cheaper, safer and more maneuverable, and how to assure the DST-guided treatment for these high-risk TB patients.

## Introduction

China has had the second highest burden of tuberculosis (TB) worldwide over the past 2 decades, causing the Chinese government to devote significant resources to the fight against TB [1]. From 1990 to 2010, the prevalence of sputum smear-positive (SS+) TB fell by 65%, the prevalence of bacteriologically positive TB declined by 48%, and the prevalence of pulmonary TB declined by 28% [2]. More than 1.3 million patients were cured under China’s national TB control program [1]. However, the emergence of multidrug-resistant tuberculosis (MDR-TB) and extensively drug-resistant tuberculosis (XDR-TB) has led to new challenges [3]. Drug resistant TB is increasingly common in China and is currently a significant public health concern. A 2007 national survey of drug-resistant TB in China estimated that 5.7% of new (incident) cases and 25.6% of previously treated cases harbored MDR-TB [4]. Thus, China has the largest absolute number of MDR-TB cases globally and perhaps the largest number of individuals who have latent infections with MDR-TB or XDR-TB [5,6,7]. Mehra estimated that by 2050, the incidence, prevalence, and mortality of MDR-TB in China will increase by 60%, 48%, and 35% respectively if an effective MDR-TB control program is not implemented and scaled-up in the near future [8].

According to the World Health Organization (WHO), the estimated case detection rate of MDR-TB was far from optimal in China where one-third of all global MDR-TB cases burden laid. In 2011 and 2013, the case detection rate of MDR-TB and Rifampicin-resistant TB (RR-TB) were only 2.6% and 7.7%, respectively [9,10]. Moreover, among detected cases with MDR-TB, fewer than 45% were cured, more than 25% were lost to follow-up, and 25% died. The heavy burden of MDR-TB, the extremely low rates of case detection and not optimal treatment outcomes of MDR-TB in China suggest that there are seriously problems in the availability, affordability, and utilization of medical care for MDR-TB. There are still huge gaps between needs, demands for the utilizations of MDR-TB medical care. From the public point of view, the undetected patients are the sources of MDR-TB epidemics; and MDR-TB patients, if fail to treatment, might further develop to the extensively or totally drug-resistant TB which is severer and fatal. Based on the previous studies, sputum smear positive retreatment patients are at high risk of developing into MDR-TB and were the source of primary transmission of MDR-TB. Understanding the accessibility of health care and planning an effective strategy would have a significant meaning in reducing the incidence of MDR-TB as well as its transmission[11,12].

The MDR-TB control program began in 2006 with the support of the Global Fund to Fight AIDS, Tuberculosis and Malaria (5th framework, GF5), and has been scaled up in China since then [13]. At present, the MDR-TB control program is implemented in all provinces and municipalities of mainland China, and prefectural TB-designated hospitals have the main roles in providing medical care to patients with MDR-TB. TB patients at high risk for MDR-TB are therefore referred from basic TB health care units to county-designated TB clinics, and then to designated prefectural hospitals for drug susceptibility testing (DST)—based diagnosis and treatment. There are five high-risk groups for MDR-TB in China who are recommended for referral: *(i)* TB patients with persistent SS+ samples, *(ii)* TB patients who are in close contact with other MDR-TB patients, *(iii)* TB patients who failed an initial standard anti-TB treatment, *(iv)* TB patients who relapsed or required retreatment, and *(v)* new TB patients without negative conversion in sputum smear microscopy after 3 months of anti-TB treatment. However, the efficacy of the recently developed MDR-TB control program is unknown. It is also unknown how many patients at high risk of MDR-TB are actually given bacteriologic-based diagnoses and in particular, the DST.

Early detection of drug resistance makes it possible to use appropriate treatment regimens[14]. Delays in the diagnosis and treatment of MDR-TB can result in patients developing persistent disease, progressive parenchyma destruction, higher bacillary loads, continuing transmission and increased mortality [14]. The purpose of the present study was to identify factors associated with access to bacteriologic-based TB diagnosis under current China’s MDR-TB control program, and to develop strategies for improving the case detection rate of MDR-TB in rural China.

## Methods

### Study sites

Eight county-/district-designated TB clinics from three provinces (3 in Jiangsu, 2 in Shandong, and 3 in Sichuan) in China were selected as study sites for the consideration of geographic and economic variation and implementation period of Global Fund and MDR-TB control program. All study sites were a part of the Global Fund-supported MDR-TB control project. Shandong and Jiangsu are plain provinces in eastern China, and they joined the Global Fund-China MDR-TB control program in July of 2010. Before implementation of Global Fund, the provincial MDR-TB surveillance system had been set up in Shandong since 2009. Sichuan is a province in western China with vast mountainous areas. It joined the program in July of 2011. In 2013, the populations of Jiangsu, Shandong, and Sichuan Provinces were 79.39, 97.33, and 81.07 million respectively, and the GDPs per capita were ¥74,607, ¥56,323, and ¥32,454 respectively.

### Data collection

SS+ TB patients who sought health care in a designated TB clinic in one of the selected sites and met the inclusion criteria (see below) were recruited from June 2012 to May 2013. Each included SS+ patient should have had at least one previous course of anti-TB treatment that lasted for longer than one month. Patients were excluded if they were sputum smear negative.

The history of previous anti-TB treatment was collected from two sources. For patients whose first episodes were before initiation of China’s DOTS program (1992 to 2005, depending on the region), information was based on self-reports. For patients whose first anti-TB treatments were after the full coverage of DOTS, treatment records were in the county TB control system and available for checking online through the national internet-based infectious disease reporting system.

Assuming that the proportion of patients receiving sputum culture testing was 70% and a confidence interval of 95%, and allowable deviation0.07, the necessary sample size was estimated as:

$$n=\frac{1.96^{2}\times 0.7\times (1-0.7)}{0.07^{2}}=165$$

All eligible subjects were face-to-face interviewed by trained clinical nurses in county-designated TB clinics using a self-reporting structured questionnaire, which collected information on demographics and socioeconomic status. The sputum culture results were from laboratories of county-designated TB clinics, and the DST results were from the laboratory of the Prefectural Centers for Disease Prevention and Control (CDCs).

### Bacteriologic diagnoses

Each enrolled patient submitted 3 sputum samples for smear and culture tests in the county-designated clinics. All SS+ patients were enrolled and all subsequent sputum samples were sent to the laboratory of the county-designated clinic for culturing. A positive culture isolate was delivered to the lab of the prefectural CDC for strain differentiation and DST following the guidelines of the Global Fund-China MDR-TB control project. The proportion method was used in the DST for isoniazid, rifampicin, streptomycin, and ethambutol [15], and the critical growth proportion for resistance was 1% for each drugs.

### Data analysis

Data were analyzed using SPSS for Windows version 17.0 (SPSS, Chicago, IL, USA). Means, medians, proportions, and 95% confidence intervals (95% CIs) were calculated as descriptive statistics. The chi-square test was used to compare subgroups regarding the probability of receiving a sputum culture test and DST. Logistic regression analysis was used to identify the significance of demographic characteristics, socioeconomic status, and disease profiles with access to sputum culture tests.

### Ethical considerations

Written consent was obtained from all participants after the study was described in the initial face-to-face interview. Approval of this study was obtained from the Ethics Committee of the School of Public Health, Fudan University (IRB: #2011-05-0308).

## Results

### General characteristics of patients

During the study period, there were 397 patients who had previously treated for TB (named as retreatment patients) registered in the study sites of the 8 counties, of whom 196 were SS+, 136 were SS-, and 65 had no sputum smear results.

All 196 SS+ patients agreed to participate in the study, and this included 117 patients from Jiangsu, 28 patients from Shandong, and 51 patients from Sichuan (Table 1). The average age was 55.14 ± 16.29 years (range: 17 to 88 years), about 78% (153/196) were male, and nearly 70% (137/196) were farmers. Most patients were illiterate (24.1%) or had less than 6 years of education (37.94%). The median (Q1-Q3) household *per capita* income was 5000 CNY (2500–9166.67 CNY), and the minimum income was 500 CNY. According to the China Statistical Yearbook of 2014, the average per capita income of rural households in China was 8895.9 CNY. Three patients (1.53%) refused to provide income information and 143 patients (72.95%) had household per capita incomes lower than the national average for rural households. There were 38 patients (19.39%) whose per capita household incomes were lower than 1500 CNY (less than $1 per day), a level of poverty first recorded in 2011.

**Table 1:** General characteristics of TB retreatment patients (n = 196) who had positive sputum smears from June 2012 to May 2013.

Variables		No.	%
Province	Jiangsu	117	59.69
	Shandong	28	14.29
	Sichuan	51	26.02
Sex	Female	43	21.94
	Male	153	78.06
Education (years)	Illiterate	47	24.1
	< 6	74	37.94
	6-	61	31.28
	9 -	10	5.13
	12 -	3	1.54
Occupation	Farmer	137	69.9
	Non-farmer	59	30.1
Household *per capita* income*	≥¥8895.9	50	25.91
	<¥8895.9	143	74.09
Previous sputum culture	Yes	30	15.31
	No	166	84.69
No. of previous TB treatments	1	161	82.14
	2	29	14.8
	3 or more	6	3.06
Last treatment outcome	Cured	126	64.29
	Failed	25	12.76
	Completed	14	7.14
	Interrupted	26	13.27
	Unknown	5	2.55

* The threshold is based on the average *per capita* income of rural households in China during 2013.

### Previous anti-TB treatments

One hundred sixty-one patients (82.1%) had one previous course of anti-TB treatment, 14.8% had 2 previous courses, and 3.06% had three or more previous courses (Table 1). The median time from the most recent anti-TB treatment to recruitment was 2.53 years (Q1-Q3: 1.02–7.73 years), with a minimum less than 1 year and a maximum of 47 years. Altogether, 15.3% of the patients (30/196) received sputum-culture testing during their previous treatments. During the most recent previous anti-TB treatment, 126 patients (64.3%) were cured, 25 patients (12.8%) failed treatment, 14 patients (7.14%) completed treatment, 26 patients (13.3%) had interrupted treatments, and data were unavailable for 5 patients (2.55%) (Table 1).

### Sputum culture testing and DST during the current treatment

**Fig 1:**
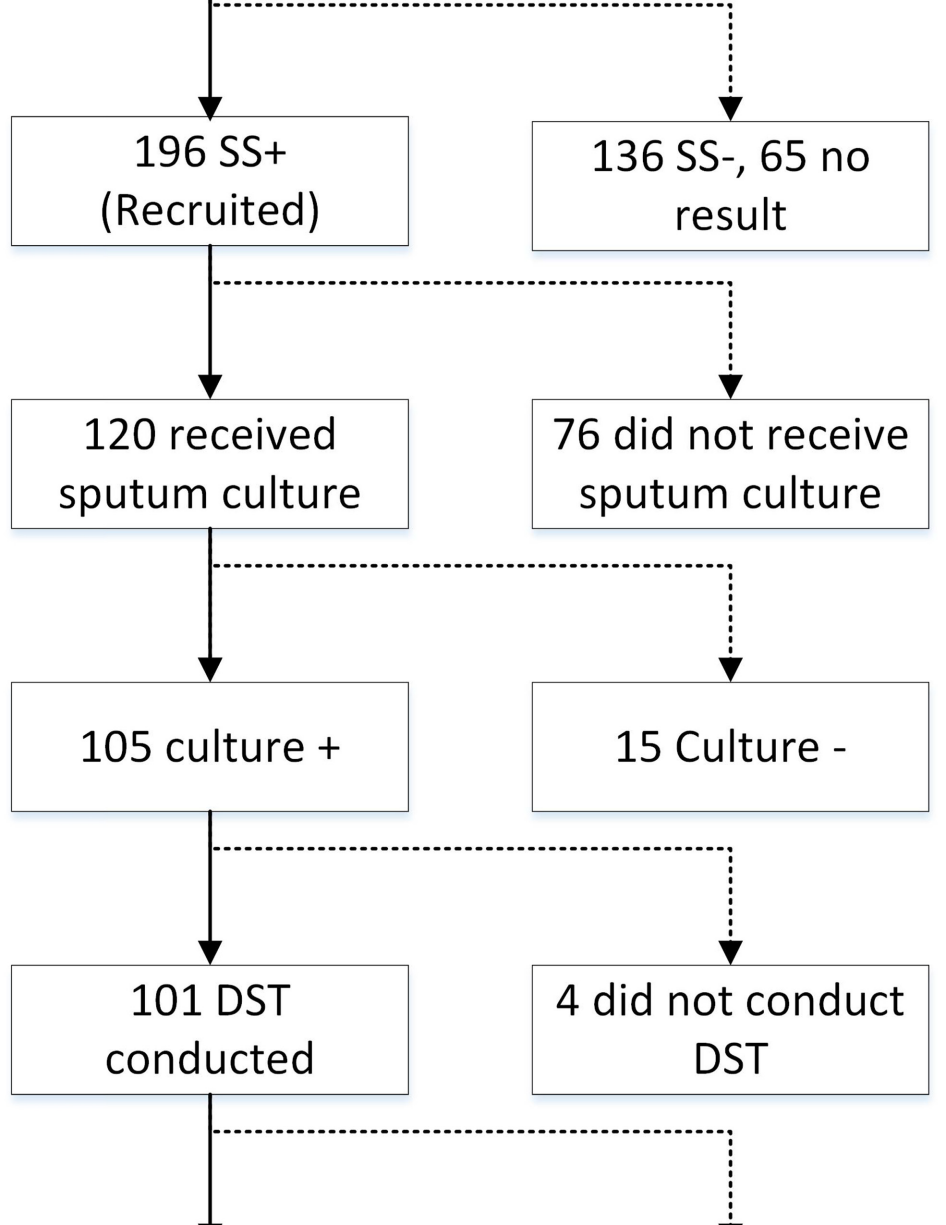
Disposition of TB retreatment patients who were initially screened (n = 397) and of recruited patients who had positive sputum smears (SS+, n = 196) from June 2012 to May 2013.

Among the 196 enrolled SS+ patients, 120 (61.2%) received sputum culture tests under the current MDR-TB control program. In total, 105 (87.5%) *Mycobacterium tuberculosis* (M. TB) isolates were collected, 101 (96.2%) were subjected to DST, and results were available for 92 isolates (Fig 1).

We analyzed the factors associated with receipt of sputum culture tests (Table 2) during the current episodes of TB. The results indicated that geographic region of residence, number of previous anti-TB treatments, household income *per capita*, receipt of a previous sputum culture, and last treatment outcome were associated with a receiving sputum culture test. In particular, patients from Jiangsu Province were more likely to receive sputum culture tests (*p* < 0.001) after referral to the MDR-TB control project. All patients with 3 or more previous treatments received sputum culture tests, but only 57.14% of patients with one previous treatment course received culture tests. Patients with household incomes *per capita* of at least 8895.9 CNY per year (the average annual income *per capita* among rural households during 2013) received more sputum culture tests than those with lower incomes (74.0% *vs*. 57.3%, *p* = 0.037). In addition, patients whose last treatment was successful had a lower probability of receiving a sputum culture test (54.3% *vs*.78.6%, *p* = 0.002). Patients who previously received sputum culture tests had a higher probability of receiving a sputum culture test during the current treatment (96.7% *vs*. 54.8%, *p* < 0.001).

**Table 2:** Factors associated with sputum culture testing in TB retreatment patients (n = 196) who had positive sputum smears.

Variable		Sputum culturing		P Value
		Yes, n = 120	No, n = 76	
		No. (%)	No. (%)	
Province	Jiangsu	96(82.05)	21(17.95)	**<0.001**
	Shandong	13(46.43)	15(53.57)	
	Sichuan	11(21.57)	40(78.43)	
Age (years)	<60	61(57.01)	46(42.99)	0.152
	≥60	59(67.05)	29(32.95)	
Sex	Female	23(53.49)	20(46.51)	0.239
	Male	97(63.40)	56(36.60)	
No. of previous treatments	1	92(57.14)	69(42.86)	**0.013**
	≥2	28(80.00)	7(20.00)	
Education (years)	Illiterate	32(68.09)	15(31.91)	0.261
	≤6	40(54.05)	34(45.95)	
	>6	47(63.51)	27(36.49)	
Household *per capita* income	≥¥8895.9	37(74.00)	13(26.00)	**0.037**
	<¥8895.9	82(57.34)	61(42.66)	
Previous culture	Yes	29(96.67)	1(3.33)	<0.001*
	No	91(54.82)	75(45.18)	
Last treatment result	Success	76(54.29)	64(45.71)	**0.002**
	Failure	44(78.57)	12(21.43)	

* Statistical analysis by Fisher’s exact test

Multivariate analysis (Table 3) showed that more patients from Jiangsu Province (OR = 24.032, 95% CI = 8.582–67.297) and Shandong Province (OR = 3.597, 95% CI = 1.113–11.692) received sputum culture tests than those from Sichuan Province. In addition, patients were more likely to receive sputum culture tests if they had 2 or more previous courses of treatment (OR = 4.984, 95% CI = 1.559–15.935), higher household income *per capita* (≥8895.9 CNY/year) (OR = 3.768, 95% CI = 1.325–10.719), and if their previous treatment was a failure (OR = 2.556, 95% CI = 1.016–6.431). Sex, age, and years of education had no statistically significant effects.

**Table 3:** Multivariable analysis of factors associated with sputum culture testing in TB retreatment patients who had positive sputum smears.

Variable	β	P Value	OR	95% CI
Province				
Shandong vs. Sichuan	3.179	**<0.001**	**24.032**	**8.582–67.297**
Jiangsu vs. Sichuan	1.28	**0.033**	**3.597**	**1.113–11.629**
Sex (Male vs. Female)	0.67	0.153	1.955	0.780–4.901
Age (Years)	0.004	0.800	1.004	0.976–1.032
Education		0.439		
≤6 years vs. Illiterate	0.543	0.310	1.722	0.603–4.917
>6 years vs. Illiterate	0.789	0.210	2.202	0.641–7.560
Number of previous anti-TB treatments	1.606	**0.007**	**4.984**	**1.559–15.935**
(≥2 episodes vs. 1 episode)				
Household income per capita (¥)	1.327	**0.013**	**3.768**	**1.325–10.719**
(≥8895.9 vs. <8895.9)				
Last treatment result (Fail vs. Success)	0.938	**0.046**	**2.556**	**1.016–6.431**

### DST result of patients with previous anti-TB treatment

There were 92 patients with DST results available for all 4 first-line anti-TB drugs (isoniazid, rifampicin, streptomycin, and ethambutol). The results indicated that 46 isolates (50.0%) had no resistance to any of these drugs (Table 4). Resistance to only 1 drug was present in 15 isolates (16.3%), and MDR was present in 28 isolates (30.4%).

**Table 4:** Resistance to first-line anti-TB drugs among isolates from TB retreatment patients with positive sputum smears for whom DST results were available (n = 92).

Resistance	Anti-TB drug	No.	%	95% CI
Susceptibility to all four first-line drugs		46	50	39.78–60.22
Resistant to single	Isoniazid	4	4.35	
drug	Rifampicin	4	4.35	
	Streptomycin	5	5.43	
	Ethambutol	2	2.17	
	Total	15	16.3	8.76–23.85
MDR	Rifampicin+Isoniazid	13	14.13	
	Rifampicin+Isoniazid+ Streptomycin	8	8.7	
	Rifampicin+ Isoniazid+ Ethambutol	1	1.09	
	Rifampicin+Isoniazid+ Streptomycin +Ethambutol	6	6.52	
	Total	28	30.44	21.03–39.84
Other drug resistance	Isoniazid+Streptomycin	2	2.17	
	Rifampicin+Ethambutol	1	1.09	
	Total	3	3.26	0–6.89

## Discussion

This study examined patients from 8 study sites in China that had implemented different period of the Global Fund supported MDR-TB control program. This program provides sputum culture tests at no charge to patients who have a high risk of MDR-TB, including TB retreatment patients who are SS+. Nonetheless, 76 of our 196 SS+ TB retreatment patients (38.8%) did not receive sputum culture tests, and only 46.9% of our high-risk patients had DST results available. These results suggest that there are serious problems in access to medical care for patients who have high risk of MDR-TB. Barriers to medical care for such patients could be identified from each step taken by people from occurrence to progress and prognosis of TB [16,17,18].

The China National TB control program provides TB diagnosis *via* sputum smear microscopy, X-ray examination, and 6–8 months of anti-TB treatment with a standardized regimen using first-line drugs at no charge. DST is not routinely performed for TB patients and is not available in most of the county/district TB clinics [19]. In 2005, China launched its MDR-TB control program, with support mainly from the Global Funds, the Gates Foundation, and national and local governments. Under this program, DST-based MDR-TB diagnosis is currently available only in the prefectural or higher levels of TB healthcare facilities.

Clinicians typically request that patients with suspected TB submit 3 sputum samples, 1 for smear testing the others for culture testing. However, some patients only provide a spot sputum for smear testing, and this quantity is inadequate for culture testing. Thus doctors often have to ask patients to return to the clinic to provide additional sputum samples for culture. Moreover, it can take nearly 2 months after submission of a sputum sample before culture results are available, because conventional sputum culturing and DST take a long time. It can also take time for transportation of isolates from the county labs to the prefectural labs.

Globally, case detection and treatment outcome of MDR-TB are the main concerns for the control of MDR-TB [20]. In the 2014 WHO Global TB report, the detection rate of MDR-TB in China was only 7.7%, far lower than in other high-burden countries such as India (57%) [10]. Poor access to sputum culture tests is the main cause for the low case detection in China. The overall sputum culture proportion of SS+ retreatment patients who are at extremely high risk of developing into MDR-TB was less than 65%, even in our study sites, which are covered by MDR-TB control program. In the numerous areas, which are not covered by any MDR-TB control programs, the performance of sputum culture and DST would be more worrying. We found that the level of regional development, the presence of a TB control system, socio-economic status, previous anti-TB treatment, and outcome from the most recent anti-TB treatment influenced patients’ access to sputum culture tests.

We also found that access to sputum culture testing varied among different geographic regions. In Jiangsu 82.1% of patients received sputum culture testing, but in Sichuan only 21.57% of patients received testing. Thus, patients from Jiangsu were 3.6-fold more likely to receive sputum culture testing than those from Sichuan. Access to TB/MDR-TB diagnosis could be affected due to the shortage of human resources and service capacity. Lack of qualified care staff and unbalanced distribution of human resource are the major factors restricting the promotion of MDR-TB control program in China [21,22]. Before implementation of Global fund, the county and prefectual TB dispensary in our study sites in Sichuan could not do culture and DTS. And Sichuan initiated the Global Funds one year later than the other 2 provinces, so there was presumably less readiness for diagnosis of MDR-TB in Sichuan. In another hand, Jiangsu and Shandong are generally more developed coastal eastern provinces, and residents generally have better economic status than Sichuan, an in-land mountainous province in western China with a lower GDP, a larger population and longer geographic distance to health services. Study has shown that a long term illness, bad health, no car ownership and long geographic distance are the barriers of getting access to health care [23]. Political and financial commitments of central and local governments are also vital for effective TB control. In China, the local government is required to provide a certain amount of the so-called counterpart funds to co-finance the project [24]. However, some provinces were unable to reach their expected goals due to insufficient resources, especially provinces in the less developed regions of western China. Access to X/MDR-TB diagnosis can also be affected by a shortage of human resources. Lack of qualified medical staff and unbalanced distribution of human resources are major factors that limit the promotion and adoption of the MDR-TB control program in China.

TB is a poverty-related disease, and poverty can be considered the cause or the result of TB [25]. Previous studies showed that patients with low socio-economic status are more likely to develop primary MDR-TB [26,27]. If not diagnosed in time, patients with MDR-TB are likely to become infectious sources for primary transmission of MDR-TB [28]. In this study, 73.0% of our patients had incomes lower than the national average, and nearly 20% had incomes lower than the poverty level. We found that patients with lower incomes had worse access to sputum culture testing. Economic factors should not be an impediment to sputum culture tests. Although the sputum culture testing is free in all program sites, poor access to the testing could result from poverty-related factors including poor health status, stigma associated with a diagnosis of TB, lack of knowledge about X/MDR-TB, and limited transportation access to testing centers [29,30].

In our study population, patients who received 2 or more courses of previous TB treatments, who had previous sputum culture tests, and whose last treatments were failures were more likely to receive sputum culture tests. This is probably because healthcare workers considered these patients to be more likely to have MDR-TB, and clinicians were more likely to be alerted to their status.

In this study, 50% of the M. TB isolates were resistant to at least one anti-TB drug, and the prevalence of MDR-TB was 30.43% among patients who received previous anti-TB treatments. This percentage of MDR-TB isolates was higher than the estimates of the WHO in 2014 (20.5%) [10] and of the National Survey of Drug-Resistant Tuberculosis in China in 2007 (25.6%) [4]. This again emphasizes the urgent need to address the MDR-TB in China and that the current situation is clearly unsatisfactory. Patients who received previous anti-TB treatments have an extremely high risk of developing MDR-TB, and are very likely sources of primary transmission of MDR-TB. Although sputum culture tests and DST are somewhat expensive, treatment of a single case with MDR-TB or XDR-TB is significantly more expensive [31]. Each patient’s status should be determined as fast as possible to prevent the spread of MDR-TB. Healthcare providers should be trained to make sure that all high risk patients have access to sputum culture tests and DST, and should not merely consider this as an optional check.

In recent years, the WHO has recognized the importance of rapid molecular TB diagnoses. Thus, a comprehensive program applying rapid diagnostic testing for TB successfully tested 84% of all SS+ patients for MDR-TB or RR-TB, and this decreased the median time needed for diagnosis from 60 days (IQR: 40–80 days) to 7 days (IQR: 5–9 days). Rapid testing can also replace smear tests and tests for rifampicin resistance with the same specimen. Use of a rapid procedure for testing TB drug resistance is important, because many patients with MDR-TB are lost to follow up or die while waiting for a diagnosis [32]. Methods such as Xpert® MTB/RIF can also greatly decrease the time before commencement of suitable treatment [33]. The application of such methods would help China to better detect MDR-TB, provided that sufficient resources are allocated [34].

### Limitations

This study examined patients from 8 counties of 3 geographically diverse provinces of China. However, compared to the more than 2600 counties in China, the sample size was small, thereby limiting the generalizability of the results. The barriers in providing medical care to patients with MDR-TB could come from the “demand side” (demographic factors, socio-economic status, limited knowledge of drug-resistant TB) or the “supply side” (limited provision of medical services, problems in policy support, financial problems, affordability of diagnosis and treatment, and capacity for providing medical care). However, the objective of the present study was to describe the receipt of bacteriological-based TB diagnosis among SS+ TB patients who were previously treated for TB, and to identify factors associated with access to bacteriologic-based TB diagnosis from the “demand side”. Thus, the present research focused on the behavior and experiences of patients.

## Conclusions

Retreatment SS+ TB patients, the high risk MDR-TB population, had poor utilization of access to bacteriologic-based TB diagnosis, which is far from optimal.

Patients from the areas which had poor economic status, shorter history of MDR-TB control program and worse health serve capacity, had less chance to get bacteriologic-based diagnosis and treatment. More training programs and capacity building courses should be provided to the health professionals before and during the implementation of the new MDR-TB control program.

Individual economic level and anti-TB treatment history were associated with the utilization of MDR-TB medical care. Pro-poor strategies need to be taken into consideration to improve the health utilization of poor TB patients especially retreatment TB patients. The next step of anti-TB strategy should be focused on how to make bacteriological-based diagnosis cheaper, safer and more maneuverable, and how to assure the DST-guided treatment for theses high-risk TB patients.
